# Tracking the eye non-invasively: simultaneous comparison of the scleral search coil and optical tracking techniques in the macaque monkey

**DOI:** 10.3389/fnbeh.2012.00049

**Published:** 2012-08-14

**Authors:** Daniel L. Kimmel, Dagem Mammo, William T. Newsome

**Affiliations:** Department of Neurobiology, Howard Hughes Medical Institute, Stanford UniversityStanford, CA, USA

**Keywords:** eye position, eye tracking, eyelink, fixation, infrared optical eye tracker, microsaccade, saccade, scleral search coil

## Abstract

From human perception to primate neurophysiology, monitoring eye position is critical to the study of vision, attention, oculomotor control, and behavior. Two principal techniques for the precise measurement of eye position—the long-standing sclera-embedded search coil and more recent optical tracking techniques—are in use in various laboratories, but no published study compares the performance of the two methods simultaneously in the same primates. Here we compare two popular systems—a sclera-embedded search coil from C-N-C Engineering and the EyeLink 1000 optical system from SR Research—by recording simultaneously from the same eye in the macaque monkey while the animal performed a simple oculomotor task. We found broad agreement between the two systems, particularly in positional accuracy during fixation, measurement of saccade amplitude, detection of fixational saccades, and sensitivity to subtle changes in eye position from trial to trial. Nonetheless, certain discrepancies persist, particularly elevated saccade peak velocities, post-saccadic ringing, influence of luminance change on reported position, and greater sample-to-sample variation in the optical system. Our study shows that optical performance now rivals that of the search coil, rendering optical systems appropriate for many if not most applications. This finding is consequential, especially for animal subjects, because the optical systems do not require invasive surgery for implantation and repair of search coils around the eye. Our data also allow laboratories using the optical system in human subjects to assess the strengths and limitations of the technique for their own applications.

## Introduction

The precise measurement of eye position is critical for the study of vision and oculomotor control, as well as related functions such as attention and oculomotor-based behavior. For over 40 years, the principal technique for measuring eye position has been the search coil, in which a small coil of wire is placed on the subject's eye either embedded in a contact lens (Robinson, 1963; Collewijn et al., 1975) or in non-human studies, surgically implanted in the sclera of the eye (Judge et al., 1980). This system offers high spatial (<1°) and temporal (<1 ms) resolution, but human subjects have difficulty tolerating the contact coils, which in addition may slip on the eye, distorting the reported position (Collewijn et al., 1975). In animal studies, use of the embedded scleral search coil comes at a particular cost, necessitating an invasive procedure to install the fine wire in the animal's eye. Moreover, the coil has a limited lifespan, often requiring additional surgical repairs and adding further delay to experimentation. Finally, as a foreign body in the eye, the coil may distort the kinematics of eye movements (Stahl et al., 2000; Frens and van der Geest, 2002).

Our goal in this study is to compare rigorously the traditional search coil technique to the latest generation of optical trackers, which track the eye optically using an infrared light source (Morimoto et al., 2000). A promising alternative to the search coil, the optical system offers the advantages of non-invasive installation and maintenance. In addition, the optical tracker measures pupil size along with position. Pupil size can reflect sympathetic arousal, thus providing a window into the emotional and cognitive state of the subject (Granholm et al., 1996; Siegle et al., 2003; Vo et al., 2008). Finally, unlike the coil, the optical apparatus is external to the eye and therefore does not interfere with the eye's natural movement.

The most obvious disadvantage of the optical system is the requirement of a clear, unobstructed view of the pupil, which is difficult to achieve in some individuals, such as those with a prominent brow ridge (especially true in rhesus macaques) or narrow palpebral fissures. In some cases, these difficulties can be overcome by retracting the soft tissue, increasing stimulus luminance to decrease pupillary size, and/or compensating for partial pupillary occlusion in software (Zhu et al., 1999). A second disadvantage is that the optical system cannot track position when the eyes are closed, creating gaps in eye position data during blinks.

Prior studies have compared the two techniques in human subjects (Yee et al., 1985; Discenna et al., 1995; van der Geest and Frens, 2002; Traisk et al., 2005), but intrinsic limitations associated with human experimentation have prevented resolution of several key ambiguities and have obstructed clear extrapolation to non-human research. In human studies, for example, the search coil is contained within a contact lens instead of surgically embedded in the sclera, which may affect coil stability and therefore contribute to known optical-coil discrepancies. In addition, human subjects are not rigidly head-fixed, and typical camera configurations do not afford the optimal, direct-angle view of the eye. Also, previous studies have not compared the ability of the two systems to detect small fixational saccades. Further investigation of these fine eye movements, including microsaccades, may yield a better understanding of covert attention, oculomotor control, saccade programming, and visual search (Hafed and Clark, 2002; Engbert and Kliegl, 2003; Engbert, 2006; Turatto et al., 2007; Hafed et al., 2009). Finally, the frame rate for optical tracking has advanced considerably since previous studies were conducted, with current systems achieving rates comparable to those typically used with the coil system.

In the present study, we compared a popular search coil system and a popular optical system in the macaque monkey, a preparation commonly used with the search coil in neurophysiological experiments. We monitored the position of a single eye simultaneously with both systems while the animal performed fixational and saccadic eye movement tasks. Simultaneous recording of a single eye allowed us to remove trial-to-trial variability as a potential source of discrepancy between the systems and attribute variation in one system to either “signal” (i.e., real movements of the eye) when it co-varied with the other system or “noise” (i.e., measurement error) when it was independent. We compared the systems in two experiments and five analyses that addressed: (1) accuracy and precision during stable fixation, (2) detection of fixational saccades, (3) measurement of instructed saccadic movements, (4) trial-to-trial drift in reported position, and (5) sensitivity to luminance change. We found broad agreement between the two systems in tracking eye position, particularly in coarse positional accuracy, sensitivity to subtle variation across trials, fixational saccade detection, and saccade amplitude. However, specific discrepancies—including elevated saccade peak velocities, post-saccadic ringing, influence of luminance change on reported position, and greater sample-to-sample variation in the optical system—warrant a needs-based consideration of the suitability of either system for the particular experimental question. The optical system, for example, is subject to a slow drift artifact caused by luminance-induced changes in pupillary diameter, which might compromise studies of known slow drift eye movements that occur during fixation (Martinez-Conde et al., 2004).

## Materials and methods

Data were obtained from two adult male rhesus monkeys (*Macaca mulatta*), C and E. Prior to experimentation each animal was prepared surgically with a head-holding device (Evarts, 1966; Adams et al., 2007) and scleral search coil (Robinson, 1963; Judge et al., 1980) made of fluoropolymer-coated stainless steel wire (model AS631 or AS632, 10- or 15-stranded, 40 or 38 gauge; Cooner Wire, Chatsworth, CA) and looped three times around the limbus before exiting the orbit (left or right eye of Monkey C or E, respectively). All procedures were in accordance with the NIH Guide for the Care and Use of Laboratory Animals.

### Apparatus

The animal was seated in a primate chair positioned within a large-volume, oscillating magnetic field 64 cm (Monkey C) or 54 cm (Monkey E) from a CRT computer monitor (38 × 29 cm, ViewSonic P225, Walnut, CA). The field coils were 114 × 104 × 107 cm, with a maximum field strength of 5 * 10^−6^ T (smaller sized fields, e.g., 50.3 × 43.0 × 47.8 cm and 2 * 10^−5^ T, generated noise in the digital camera signal). Eye position was monitored with both a scleral search coil and optical system simultaneously. With the animal's head stabilized, the optical system apparatus was mounted on the chair using custom hardware. Specifically, we positioned a “hot mirror” that reflects IR light but transmits visible light (Edmund Optics, Barrington, NJ) at a 45° angle with respect to vertical in front of the animal's eyes, with a notch cut to accommodate the snout. The mirror was sufficiently large (12.7 × 5.1 cm) that its margins were outside the animal's view of the monitor. We positioned an IR camera above the animal pointing downwards toward the mirror. This design allowed the camera to view the eyes in the infrared band (as reflected by the mirror) along an optical path (~38 cm) parallel to the animal's view of the computer monitor in the visible band (through the mirror). We optically recorded from the coil-implanted eye and centered the camera's field of view on the studied eye. An IR illuminator positioned above the animal and just adjacent to the camera's optical path was directed into the mirror and trained on the recorded eye at a path length of ~15 cm.

Behavioral reinforcement in the form of liquid rewards was delivered via a gravity-fed juice tube placed just inside the animal's mouth; fluid flow was regulated by a computer-controlled solenoid valve. Stimulus presentation, behavioral control and analog recording were managed by Apple Macintosh G5-based computers (Cupertino, CA) running custom scripts for the EXPO software package written by Peter Lennie (University of Rochester, NY) with modifications by Julian Brown (Stanford University, CA).

For the purposes of real-time behavioral control, we used the eye position reported by either the coil (Monkey C) or optical system (Monkey E); the latter had the advantage of forcing repetition of trials in which the animal blinked. For the luminance-step task (see below), we exclusively used the coil system for behavioral control so that any spurious deviations in reported eye position by the optical system could be measured without aborting the trial.

Stimulus luminance was measured with the PR650 SpectraColorimeter (Photo Research, Chatsworth, CA) from behind the hot mirror at the position of Monkey E.

### Search coil settings and online calibration

Output from the search coil was fed into a current demodulator that transformed the signal into an analog voltage approximately linearly proportional to eye position in separate horizontal and vertical channels that were sampled at 1.38 kHz by the EXPO software, which stored the data to disk for offline analysis. The field coil and current demodulator, models RZPWDR-U and RZPHDT-U, respectively, were manufactured by C-N-C Engineering (Seattle, WA). The field coil frequency was adjusted such that both channels were at “Resonance.” The single-pole RC filter on the demodulator input was adjusted to a time constant of 0.5 ms. Before each day of experiments, we calibrated the *Gain*, *Offset*, and *Phase* (or linear scale, translation, and rotation, respectively) adjustments of the coil system in a separate task using a set of five fixation points (*FP*), including the origin, two horizontal (±16°), and two vertical (±12°) target positions.

### Optical tracker settings and online calibration

In addition to the coil system, we simultaneously tracked eye position and pupil area using an infrared optical eye tracking system, EyeLink 1000 by SR Research (ON, Canada), which included a high-speed (up to 2000 frames per second) IR camera, illuminator, and proprietary software running on a custom workstation (*Host PC*). For Monkey C, we configured the eye tracking software to track the center-of-mass of the pupil (*Centroid* mode), which we found to exhibit less sample-to-sample noise, but was more susceptible to errors caused by partial occlusion of the pupil by the eyelid. We encountered more pupil occlusion problems in Monkey E, and we therefore used the *Ellipse* tracking mode for this animal, which computes the center of an elliptical fit to the pupil, mitigating issues of pupil occlusion. For both animals, we increased the computer monitor background luminance, thereby decreasing pupil size and reducing pupil occlusion effects even further (see Table 1 for a summary of data acquisition and analysis details for each animal). In both animals, the software incorporated the position of the corneal reflection in estimating eye position (*Pupil-CR* mode; Merchant et al., 1974), a setting we found produced the least noise. We captured the eye position at a frame rate of 1 kHz in head-referenced coordinates (*HREF* mode) appropriate for determining gaze in a head-restrained subject.

**Table 1:** **Data selection and processing for Monkeys C and E**.

	**Monkey C**	**Monkey E**
Pupil fitting method	Centroid	Ellipse
Tasks analyzed	Instructed saccade (IS)	IS, luminance-step
Window radius FP/target	4/4°	1.5/2.5°
Target eccentricities	1–8°	1–32°
Baseline filter for all analyses	Low-pass 4^th^-order Butterworth (475 Hz); low-pass RC/RC^*a*^ (318 Hz) for coil/optical	
Filter for accuracy and precision analysis	Heuristic^*a*^/heuristic for coil/optical	–
Filter for fixational and instructed saccade analyses	Heuristic^*a*^/heuristic for coil/optical	Heuristic^*a*^ for coil and optical
Source of data for offline calibration	IS fix (0°) and hold (8°) periods	Nine-point fixation task and IS fix period (0°)
FPs/targets excluded from accuracy analysis	0, 8°	0°
Saccade detection threshold multiplier (λ)	6	10

a Our own implementation of a proprietary filter (see Materials and Methods, Offline Signal Processing).

For real-time visualization and behavioral control, we calibrated the optical tracker before each day's experiments using the included *HV9* routine that applies a proprietary non-linear, biquadratic transformation to the raw position signal (see below for offline calibration procedure used for data analysis). The routine required the animal to fixate nine FP's positioned in a 3 × 3 grid with 12° between each row and 16° between each column. These targets included the five used for coil calibration in addition to four corner targets that were not necessary for online coil calibration because the coil hardware lacked additional adjustments for corner positions. Target and background luminance were matched to those in the instructed saccade task, since estimation of eye position may depend on pupil size (and hence stimulus luminance) for optical systems (Stahl et al., 2000). We then applied a second stage of linear gain and offset adjustments to the analog output of the Host PC that served to transform the position signal to that expected by the EXPO system, which then sampled the analog output at 1.38 kHz. All offline analysis of the optical signal was performed on the raw, uncalibrated position signal that was captured and maintained as digital samples on the Host PC (see below).

For all behavioral tasks, a digital pulse was broadcast from the EXPO system to the Host PC at a fixed time in each trial and served to synchronize the event and eye position data between the two systems. In addition, trials in which a blink was detected by the optical system were excluded from analysis.

### Experiment 1: instructed saccade task

**Figure 1:**
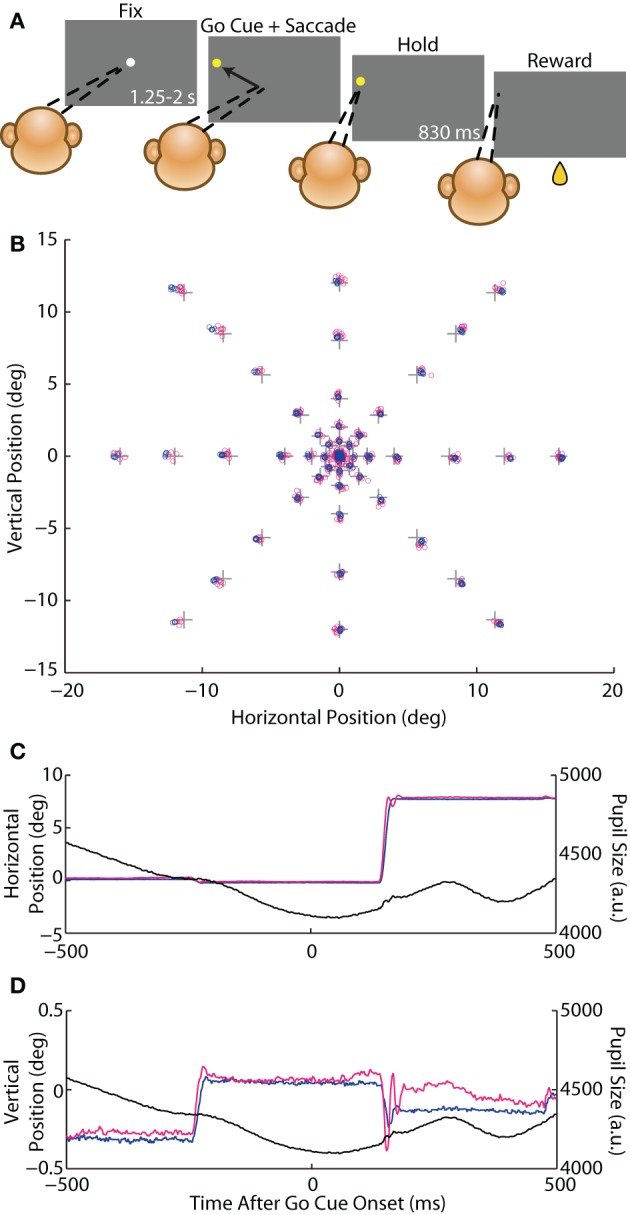
**Instructed saccade task. (A)** Schematic of the behavioral task. A rhesus macaque fixated (*dashed lines*) a white circular fixation point (FP) for 1.25–2 s (*fix* period) and was then presented with a yellow peripheral target that served as the *go cue* to execute a *saccade* to that target. The animal fixated the target for 830 ms (*hold* period) and received a juice *reward*. **(B)** *Crosses* represent the central FP and 46 possible saccade target locations. Average eye position from the fix and hold periods from each trial from Monkey E are overlaid as *circles* for the coil (*blue*) and optical (*magenta*) systems. **(C,D)** Example traces of an 8° rightward saccade from Monkey E showing horizontal **(C)** and vertical **(D)** eye position, as well as pupil size (*black*), for the coil (*blue*) and optical (*magenta*) systems plotted against time after the go cue.

Prior to experiments, we trained the animals on an *instructed saccade* task (Figure 1A). A given trial began with the appearance of a white, circular FP (diameter 0.5°) displayed on a dim gray background (20% *grayscale* or 20% of each of the red, green, and blue channels; luminance 30 cd/m^2^ with FP in place and luminance 12 cd/m^2^ with background only). The gray, instead of black, background served two purposes: (1) to increase the overall stimulus luminance so as to reduce the pupil size and any obstruction of the pupil margin that might occur with a larger pupil; and (2) to reduce the change in luminance when the FP was acquired, and thereby reduce the change in pupil size, which may contribute to spurious measurement of eye position with the optical system (see Luminance-Step Task). To initiate the trial, the animal acquired the FP by fixating his gaze within an invisible, circular *fixation window* around the FP of radius 4° (Monkey C) or 1.5° (Monkey E). After maintaining fixation for a specified interval (*fix* period, uniformly distributed from 1.25 to 2 s), a peripheral yellow *target* appeared (diameter 0.5°; 100% of the red and green channels; total screen luminance 27 cd/m^2^) and the FP simultaneously disappeared, cueing the animal to execute an *instructed saccade* to the target within 2 s. After maintaining fixation within a *target window* of 4° (Monkey C) or 2.5° (Monkey E) around the saccade target for 830 ms (*hold* period), the target was extinguished and the animal was rewarded with a drop of juice. The FP for the next trial was presented after a 2 s inter-trial-interval (*ITI*). If the animal failed to maintain fixation for the fix or hold periods or failed to make the instructed saccade, the trial was immediately aborted and the task entered the ITI. On each trial, we randomly selected the target location from a set of possible locations, while the FP location remained constant within a block of trials. This design allowed us to measure larger, stimulus-directed saccades used to acquire the target as well as static fixational accuracy and smaller, fixational saccades that occurred during the fix and hold periods.

We varied the positions of both the FP and the target relative to the FP so as to maximize the range of measured saccade amplitudes, directions, and orbital positions. Each day of experiments included a block of trials with the FP at the center of the monitor positioned such that the animal's eye was approximately centered in the orbit when fixating the FP (defined as [0,0°]). We varied the relative target position across eight directions (0–315° in increments of 45°) and six amplitudes (1, 2, 4, 8, 12, and 16°) for a total of 46 target locations (Figure 1B)—monitor dimensions did not accommodate vertical 16° targets. In an additional block, we positioned the FP in the top-right corner of the monitor ([15,11°]) with targets that spanned three directions (180, 225, and 270°) and three amplitudes (16, 24, and 32°), or 8 target locations (in this configuration, downward 32° targets exceeded the monitor dimensions). These *corner* data were included in analysis of saccade kinematics.

### Experiment 2: luminance-step task

In early experiments, we observed a possible interaction between pupil size and eye position in the optical measurements, such that changes in pupil size were mistakenly reported as changes in position. We decided to measure this interaction directly by inducing changes in pupil size (by changing stimulus luminance) while the animal maintained fixation of a static FP (Figure 8B). In the *luminance-step* task, the animal was required to fixate a central, circular FP (0.5° diameter) displayed against a uniform background. Following a specified fixation interval (*pre-step* period, uniformly distributed from 2 to 3 s), the luminance of the display was changed abruptly (*step*) by varying either the background luminance (*vary-BG*) or the luminance of the FP (*vary-FP*) while the FP location remained fixed. The animal was required to fixate for an additional 2–3 s (*post-step* period), after which a reward was delivered. Before presenting the FP for the next trial, we imposed an 830 ms ITI, during which the background color was updated to that of the next trial, thus providing additional time for the pupil to adjust to the new luminance.

The vary-BG and vary-FP versions of the task were carried out in separate blocks. In the vary-BG version, which produced the greatest changes in luminance, the trial began with a pre-step background of either white (74 cd/m^2^) or black (8 cd/m^2^). At the step, the background color changed to one of five possibilities: 0 (black), 25, 50, 75, or 100% (white) grayscale (note that the background remained constant in the pre-post cases of white–white and black–black). During the pre-step period, the animal fixated an FP of 50% grayscale. The FP luminance remained constant during the post-step period for most conditions. However, when the background changed to 50% grayscale (which would have rendered the FP invisible!), the FP was simultaneously changed to either 0 or 100%. This experiment thus incorporated six post-step conditions (two FP luminance conditions at the 50% background, and one condition for each of the remaining four backgrounds) with the following total luminance values, including both the FP and the background: 8, 21, 29/50, 57, or 74 cd/m^2^ (the “29/50” split accounts for the two different FPs used with 50% background). In all, the two pre-step conditions and six post-step conditions accounted for 12 total conditions. (All luminance values represent total screen luminance with the appropriate FP in place, not the luminance of the background alone).

The vary-FP version produced a more subtle change in luminance perhaps more akin to what a subject would experience when transitioning from one target to another. Throughout the task, the background remained black (0% grayscale). During the pre-step period, the FP was either 20 or 100% grayscale (2 or 18 cd/m^2^, respectively). At the step, the FP changed to one of five possibilities: 20, 40, 60, 80, or 100% grayscale (2, 6, 10, 14, or 18 cd/m^2^, respectively), while the background remained black. The two pre-step conditions and five post-step conditions made for 10 total conditions.

The 12 or 10 conditions for the vary-BG or vary-FP tasks, respectively, were presented with equal frequency and randomly interleaved.

### Offline signal processing

At the outset of our study, we faced the question of how to compare the coil and optical systems. Should we compare them in their native, “out-of-the-box” forms as a basic end-user would experience them? Or should we instead attempt to equalize offline the various filtering and calibration procedures employed by either system? We opted for the latter approach in the present analysis, reasoning that having applied all known processing procedures equally to both systems, any differences we then uncover would reflect the “intrinsic” nature of the two measurement techniques and would likely be experienced by even sophisticated end-users. However, we point out below the rare instances when the equalization of processing changed meaningfully the results one would obtain from using either system out-of-the-box.

For all offline analysis, eye position data recorded by the two systems were low-pass filtered using a fourth-order Butterworth filter with a cutoff frequency of 475 Hz, or 95% of the Nyquist frequency of the slower system. In addition, the coil system applies a broad, low-pass, single-pole RC filter (τ = 0.5 ms or −3 dB at 318 Hz) at the input stage of the demodulator, which we replicated in software and applied to the optical signal.

The optical system optionally applies a proprietary heuristic filter in software that reduces sample-to-sample variation during fixation and is recommended by the manufacturer. In brief, the filter removes one-sample “spikes” and two-sample “pulses” in eye position data, which reduces the false positive rate in detecting small-amplitude saccades. We attempted to replicate the filter in software using a published description on which the proprietary filter is based (Stampe, 1993). We shared our implementation with the manufacturer who confirmed that we had “captured the essence of the routines” (Pers. Comm.). Having our own implementation allowed us to apply the filter equally to data from both the coil and optical systems, which we did for saccade analyses in Monkey E for whom the optical data was collected without the proprietary filtering (Table 1). (We did not apply the heuristic filter to accuracy and precision analyses in Monkey E because they were designed explicitly to measure variation of eye position during fixation. However, in a separate step, we did apply the filter solely for the purposes of detecting fixational saccades that could exclude a trial from the accuracy and precision analyses). Data from Monkey C was collected with the proprietary filter applied irreversibly to the optical data (“Extra” mode), and we subsequently applied our own heuristic filter to the coil data for all analyses from Monkey C.

### Offline calibration

As described above, we collected linearly calibrated eye position signal from the coil system as well as the raw, uncalibrated position data from the optical system. As mentioned, the optical system applies a proprietary biquadratic calibration algorithm online (that does not influence the raw offline signal), which we found to be more accurate than standard linear calibration. We replicated the proprietary algorithm using published methods on which the algorithm is based (Sheena and Borah, 1981), and confirmed the similarity of the two algorithms both with the manufacturer (Pers. Comm.) and through our own tests. Offline, we applied the biquadratic calibration separately to the linearly-calibrated coil and raw optical signals (note that the biquadratic calibration includes linear terms, listed below, that replicate the linear calibration already applied to the coil signal). Unlike standard linear calibration, the biquadratic approach compensates for non-linearities in reported position along the horizontal or vertical dimensions (i.e., rectification), interactions between the horizontal and vertical channels (e.g., rotation), as well as saturating nonlinearities specific to each corner. To compute the calibration parameters, we related the expected horizontal and vertical eye positions, *x*_*k*_ and *y*_*k*_, respectively, at fixation target *k* to the reported position signals *X*_*k*_ and *Y*_*k*_ as,

(1) $$x_{k}=a+bX_{k}+cX_{k}^{2}+dY_{k}+eY_{k}^{2}+m\left(q\right)X_{k}Y_{k}$$

(2) $$y_{k}=f+gX_{k}+hX_{k}^{2}+iY_{k}+jY_{k}^{2}+n\left(q\right)X_{k}Y_{k}$$

We first solved for parameters *a* to *e* and separately *f* to *j* (omitting *m* and *n*) using five fixation targets (two vertical, two horizontal, and one central); given five equations (one for each target location per dimension) and five free parameters for each equation, we obtained a unique solution. We then applied this partial calibration to the reported position signals from the four corner fixation targets and solved for *m*(*q*) and *n*(*q*) separately for each corner. For the purposes of calibration, the reported position signals were taken as the mean signal during the final 300 ms of the fixation period and then averaged across repeated presentations of the same fixation target (see below for choice of fixation periods and targets).

The selection of fixation targets for calibration differed by animal. For Monkey E, we ran a separate fixation task at eight peripheral targets in the HV9 arrangement described above, requiring fixation for 750 ms on each target and at least five successful fixations per target. For Monkey C, we observed severe saturation of the coil-reported signal at targets ≥12°, and so were unable to use the separate fixation task for calibration. Instead, for both coil and optical systems, we extracted the reported signal from the fix period (central FP) and hold period of saccade targets at 8° eccentricity during the instructed saccade task. As a result, we restricted our analysis of Monkey C to saccade targets ≤8°. (Note that using the 8° targets for calibration artificially lowered the measured error at these targets, but did not influence our analysis of precision, i.e., consistency of measured position across trials). Finally, for Monkey E, we observed a slight difference in reported position at the central FP between our pre-experimentation calibration routines and the instructed saccade task. We attributed this difference to the insufficient number of trials at the central FP during the calibration routine. To compensate, we computed calibration parameters using fixation data from the calibration routine for peripheral FP's, but from the fix period of the instructed saccade task itself for the central FP. While this artificially lowered the absolute error we measured at the central FP, it served more importantly to prevent a simple translation of the entire visuomotor field from contributing to error at all peripheral targets, presumably a more critical measurement. Targets from the instructed saccade task that were used for calibration (8° for Monkey C and central FP for both animals) were excluded from the accuracy analysis (Table 1).

### Saccade detection

We detected saccades based on the eye velocity profile smoothed over five samples as described previously (Engbert and Kliegl, 2003). Briefly, we computed velocity thresholds for the horizontal and vertical dimensions as a multiple λ of the variance of velocity for the respective dimension within each trial (Monkey C λ = 6, Monkey E λ = 10; for a given animal, the same value of λ is applied to both systems and both dimensions, though the values for horizontal and vertical variance may differ). The thresholds took the form of an ellipse such that the velocity criterion depended on both the magnitude and direction of eye velocity. Eye movements that exceeded this threshold for a minimum duration τ (5 ms, both animals) were scored as a saccade. We occasionally observed several small saccades in rapid succession. We assumed noise in the position signal was responsible for breaking a single saccade into two or more physiologically improbable “sub-saccades.” Therefore, we combined any two sub-saccades occurring within 30 ms of each other into a single saccade for analysis purposes (Horwitz and Albright, 2003; Kimmel and Moore, 2007).

Naturally, any comparison of saccade detection will depend critically on the parameters used for the detection algorithm. For a given animal, we systematically varied the λ and τ parameters (again with the constraint that λ and τ were constant across systems and, in the case of λ, across dimensions within an animal) and chose the combination that maximized the number of fixational saccades detected by both tracking systems (which we assumed to represent true saccades) while simultaneously minimizing the number detected by only one system (assumed to largely represent false positives). An expert human observer manually inspected the saccade detection performed on all individual traces to confirm the suitability of the algorithm and parameters.

### Accuracy and precision analysis

Comparisons of accuracy and precision between the two eye tracking systems were based on eye position in the final 300 ms epoch of the fix and hold periods of the instructed saccade task with a central FP. Epochs during which a saccade with amplitude greater than 0.5° was detected were excluded from analysis; for analysis of sample-to-sample variation (see below), epochs with a saccade of any size were excluded. We used a repeated measures ANOVA design to compare the accuracy (deviation from intended target) or precision (across- or within-trial variation of eye position) between the two systems, a design that harnessed the pairwise simultaneity of the recordings. Tracking system served as a within-subject factor, while target direction (categorical variable) and eccentricity (continuous covariate) both served as between-subject factors. *Post-hoc* pairwise *t*-tests (Sidak-corrected for multiple comparisons) revealed any differences between the systems at specific target eccentricities or directions. Analyses were performed separately for the two animals.

## Results

Here we report data from two experiments (instructed saccade and luminance-step tasks) and five analyses (accuracy and precision, fixational saccade detection, saccade metrics, trial-to-trial drift, and luminance/position interaction). The first four analyses were performed on data from Experiment 1 while the last derives from Experiment 2. For each animal, we selected for analysis the experimental session with the best calibration for both the optical and search coil systems. Experiment 1 included 320 trials from Monkey C and 466 trials from Monkey E using a central FP position, with an additional 82 trials from Monkey E using an eccentric corner FP to permit measurement of large-amplitude saccades. For Experiment 2, we collected 185 trials from Monkey E in a separate session on the luminance-step task.

### Experiment 1, analysis 1: accuracy and precision

**Figure 2:**
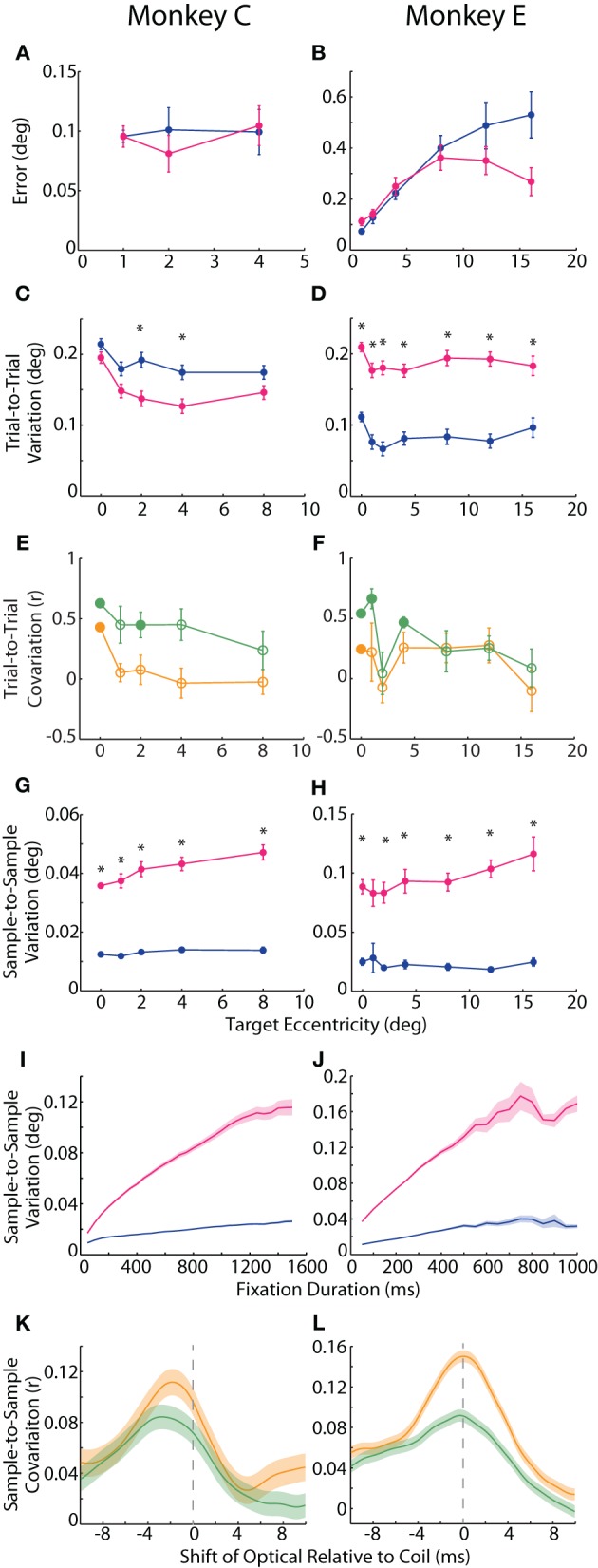
**Accuracy and precision of static fixation. (A,B)** Accuracy was measured as the average error, or distance of the average hold-period eye position (across trials) from the intended target. Plotted is the mean error across targets of the same eccentricity. **(C,D)** In one measure of precision, *trial-to-trial variation*, we computed the difference in fix- or hold-period eye position on each trial from the mean fix- or hold-period position across trials for each target. Mean trial-to-trial variation across targets was then plotted against target eccentricity. **(E,F)** The correlation between the two systems in absolute hold-period eye position across trials to the same target was computed separately for horizontal (*orange*) and vertical (*green*) dimensions, and the mean correlation coefficient across targets of the same eccentricity is plotted (except for the central fixation point (FP), for which a single value is plotted based on fix-period position). *Filled circles* represent significant correlations for the central FP or mean values significantly different from zero for peripheral targets (*p* < 0.05, *t*-test, Sidak-corrected). **(G–J)** In the second measure of precision, *sample-to-sample variation*, we computed the variation in fix- or hold-period position from sample to sample, shown for a 300 ms window at different target eccentricities **(G,H)** and at the central FP for different temporal windows **(I,J)**. **(K,L)** Samples from 50 ms epochs from the fix period were correlated between the two systems. For a given fixation epoch, samples from the coil system were held constant, while earlier (negative shift) or later (positive shift) samples from the optical system were used to compute the correlation coefficient, which was then averaged across fixation epochs to construct the cross-correlograms shown for the horizontal (*orange*) and vertical (*green*) dimensions. *Asterisks* represent significant differences between the two systems at a given target eccentricity (*p* < 0.05, paired *t*-test, Sidak-corrected). Error bars or shading represent SEM. Data from Monkeys C and E shown in the left and right columns, respectively. Colors in **A–D**, **G–J** as in Figure 1.

We defined accuracy to be the positional error at each target—the distance between reported eye position (averaged across trials for each target) and target position. In Figure 1B, one can appreciate this metric as the proximity of a cluster of trial-averaged fixation positions to their intended target. We plotted the mean error across targets of the same eccentricity in Figures 2A,B. For Monkey E, error increased with target eccentricity for both systems (*F* = 48.3, *p* < 10^−7^), although this error never exceeded 0.6°. Overall, error was greater in the coil than optical systems (*F* = 7.6, *p* < 0.01), and this difference increased with eccentricity (*F* = 32.2, *p* < 10^−5^). The difference between systems also depended on target direction (*F* = 9.7, *p* < 10^−6^), with upward (*p* < 0.005) targets being worse for the optical system and leftward (*p* < 0.008) targets being worse for the coil; however, differences at these specific directions were not consistent across additional datasets. In Monkey C, we limited all analyses to targets of 8° or less due to extreme saturation of the coil signal that we believed was not intrinsic to the system itself (unfortunately, additional corrective experiments were not possible in this animal). Across this more limited range, error did not differ between the systems or with eccentricity or direction for Monkey C. In the analysis of accuracy for both animals, we excluded data from the central FP because they were also used for calibration; for Monkey C, we additionally excluded the 8° targets for this same reason (see Materials and Methods).

We considered two different measures of precision. For repeated fixations of a given target, both tracking systems reported some variation in eye position within the permitted window. This variation manifested in the trial-averaged eye position across repeated fixations (*trial-to-trial variation*) as well as in the deviation across samples within a given trial (*sample-to-sample variation*), discussed below. One can observe trial-to-trial variation as the area, or spread, of a given cluster in Figure 1B. To compute trial-to-trial variation, we determined the average across-trial eye position for a given target and then, for each trial, calculated the distance from the reported position on that trial to the average position. This distance, averaged across trials, provided a measure of trial-to-trial variation for each target. In our analysis, we found opposite effects in the two animals, with the coil system demonstrating higher variation in Monkey C (*F* = 9.54, *p* = 0.002), but lower variation in Monkey E (*F* = 146, *p* < 10^−29^) compared to the optical system (Figures 2C,D). We confirmed this trend in additional experimental sessions, including ones using the alternative centroid tracking method for Monkey E. Note that the primary difference between the animals was an increase in coil-reported variation from Monkey E to Monkey C, suggesting that a sub-optimal property of the coil peculiar to Monkey C may have been responsible for the discordance between animals.

The across-trial variation was likely due to both variation in actual eye position (“signal”) and measurement error (“noise”). We attempted to place a lower bound on the signal-based variance by correlating, for a given target, the average eye position on each trial between the two systems, reasoning that the correlated portion should represent true variation. We correlated the signed horizontal and vertical positions separately, thus generating two correlation coefficients for the central FP and each target. Here we focus in particular on the correlations at the central FP because all trials contributed to this analysis, whereas correlations at the peripheral targets were limited to the roughly eight trials at each target and are therefore less reliable. At the central FP, horizontal (H) and vertical (V) positions were significantly correlated across trials for both Monkeys C (*r*_*H*_ = 0.43, *p*_*H*_ < 10^−16^; *r*_*V*_ = 0.63, *p*_*V*_ < 10^−38^) and E (*r*_*H*_ = 0.25, *p*_*H*_ < 0.001; *r*_*V*_ = 0.54, *p*_*V*_ < 10^−17^). We also plot mean *r* values across peripheral targets of the same eccentricity in Figures 2E,F. For peripheral targets, we found that the systems were significantly correlated at some eccentricities (filled circles indicating mean coefficient was significantly different than zero by *post-hoc t*-test, Sidak-corrected). Additionally, for Monkey C, trial-to-trial variation was more correlated in the vertical than horizontal dimensions (*F* = 9.8, *p* = 0.005); in Monkey E, we observed the same trend of greater vertical correlations at some eccentricities, although the difference was not significant overall. The degree of correlation did not depend linearly on target eccentricity.

In our second measure of precision, we quantified the sample-to-sample variation as the median distance between each sample of a given fixational epoch and the average eye position during that epoch (where an epoch was defined as the final 300 ms of either the fix or hold periods, and epochs with fixational saccades of any size were excluded—see Materials and Methods). This variation is observable as the overall waviness of the eye position signal about its mean in the timecourses of Figures 1C and especially 1D. Though the absolute magnitude of this variation was relatively small (rarely exceeding 0.1°), we found roughly three times greater sample-to-sample variation in the optical system for both animals (Monkey C: *F* = 200.6, *p* < 10^−37^; Monkey E: *F* = 82.0, *p* < 10^−13^), as shown in Figures 2G,H. This difference was significant (*p* < 0.001) at nearly all target eccentricities by *post-hoc* pairwise analysis (except for Monkey E at 1°, *p* = 0.08). In addition, sample-to-sample variation increased with increasing eccentricity overall (Monkey C: *F* = 15.0>, *p* < 0.001; Monkey E: *F* = 8.1, *p* = 0.005), though this trend was more pronounced for the optical system (Monkey C: *F* = 10.5, *p* < 0.001; Monkey E: *F* = 9.8, *p* = 0.002). Sample-to-sample variation did not depend on target direction.

In addition to high-frequency variation from sample to sample, we noticed that the position signals at times underwent slower changes over a longer timescale (e.g., Figure 1D optical trace from 250 to 500 ms). To capture variation across a range of timescales, we varied the duration of the temporal window in which we computed sample-to-sample variation (Figures 2I,J). At all durations and for both animals, the optical traces varied more than those from the coil system (Monkey C: *F* = 78.0, *p* < 10^−8^; Monkey E: *F* = 42.3, *p* < 10^−5^). With both systems, variation increased with increasing duration (Monkey C: *F* = 949.9, *p* < 10^−22^; Monkey E: *F* = 117.1, *p* < 10^−8^), though it increased at an even greater rate in the optical system (Monkey C: *F* = 821.6, *p* < 10^−21^; Monkey E: *F* = 137.6, *p* < 10^−9^). The increase in variation with duration implied that the eye position signal varied at multiple timescales: fast variation that was detected in short epochs and slower variation revealed in longer epochs. For both systems, the variation vs. duration relationship began to saturate with longer durations, suggesting that the present fixation durations captured most of the variation one would expect for even extended periods of fixation.

As with trial-to-trial variation, we attempted to separate signal from noise in our measure of sample-to-sample variation by correlating position signals. We were careful to remove large changes in eye position that would artificially increase our measure of correlation, and so we divided the entire fix period (central FP only) into epochs of static fixation and fixational saccades. From each epoch of static fixation, we extracted 50 ms of eye position samples from the coil system and correlated the samples with the same period from the optical system. To allow for slight temporal asynchronies in aligning the systems, we computed a cross-correlogram by shifting the window from the optical system in 1 ms increments (up to ±10 ms) and re-estimating the correlation at each shift, plotting the average correlation coefficient across epochs in Figures 2K,L. (Note that the original sampling rates, 1.38 or 1 kHz, supported shifts at this temporal resolution). To pair samples from two signals sampled at slightly different rates, we employed a standard signal processing technique in which we upsampled the coil and optical data to 11.024 kHz and 11 kHz, respectively, and then applied a low-pass smoothing filter at just below the Nyquist frequency of the original sampling rate (Butterworth, 475 Hz cutoff) before computing correlations (Lyons, 1997). We found that the systems were weakly but significantly correlated in both absolute horizontal and vertical positions for both Monkeys C (peak correlation coefficient: *r*_*H*_ = 0.11, *p* < 10^−24^; *r*_*V*_ = 0.084, *p* < 10^−16^) and E (*r*_*H*_ = 0.15, *p* < 10^−131^; *r*_*V*_ = 0.092, *p* < 10^−49^). The correlations peaked when optical samples were taken 2.3 or 0.1 ms (Monkey C or E, respectively) before those of the coil. We attributed this temporal shift to delays in the synchronization pulse from the Expo system to the EyeLink Host PC; we used these shift values in the saccade onset analysis below to compensate for any temporal delays.

### Experiment 1, analysis 2: fixational saccade detection

A challenge for any tracking system is the detection of small-amplitude saccades that occur during fixation (e.g., Figures 1C,D at −250 ms), as these eye movements can be of comparable amplitude to noisy signal fluctuations introduced by the measurement technique. We applied a saccade detection algorithm (Materials and Methods) to data obtained from both tracking systems during identical fix periods, considering a single saccade to be detected by both systems when the start times reported by the two systems were within 30 ms of each other. A given saccade could be paired with at most one saccade from the other system.

**Figure 3:**
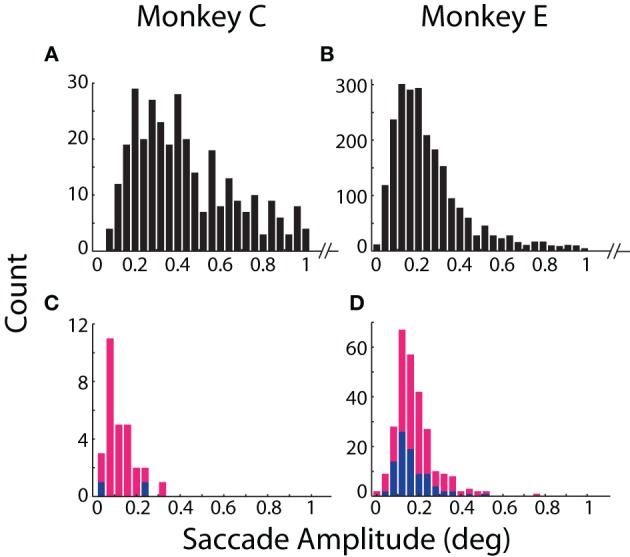
**Detection and amplitude of fixational saccades.** Distributions of fixational saccade amplitude are shown separately for **(A,B)** saccades mutually detected by both systems and **(C,D)** saccades detected only by the coil (*blue*) or optical (*magenta*) system. Note differences in scale of the ordinate between mutually **(A,B)** and exclusively **(C,D)** detected saccades. Distributions in **(A,B)** are truncated at >1° for emphasis on smaller movements. All saccades >1° (89 in Monkey C; 192 in Monkey E) were detected by both systems. Data from Monkeys C and E shown in the left and right columns, respectively.

Our results revealed general agreement in the sensitivity of the systems to fixational saccades (Table 2). The two systems jointly detected the vast majority of fixational saccades detected independently by one or the other system, for both Monkeys C (93%) and E (90%). The amplitudes of the jointly detected saccades, as measured by the coil system, ranged between 0.15 and 2.4° in Monkey C and between 0.04 and 1.6° in Monkey E (2.5th and 97.5th percentiles, respectively), as partially shown in Figures 3A,B. (Monkey C was afforded a larger fixation window around the FP, tolerating larger fixational saccades). The optical system missed 0.49 or 3.5% of saccades that were detected by the coil system, while the coil system missed 6.2 or 6.8% of saccades detected by the optical system for Monkey C or E, respectively (Figures 3C,D). We compared the amplitudes of the missed saccades for Monkey E, for whom we had sufficient number in both systems. We found that the amplitudes of the saccades detected only by the optical system (median = 0.17°) were slightly but significantly greater (Mann–Whitney, *p* = 0.03) than those detected only by the coil system (median = 0.15°), suggesting the coil system may be capable of detecting slightly smaller saccades.

**Table 2:** **Number of fixational saccades detected by either or both systems**.

		**Coil**	
		**Detected**	**Not detected**
**MONKEY C**			
Optical	Detected	409	27
	Not detected	2	N/A
**MONKEY E**			
Optical	Detected	2483	180
	Not detected	89	N/A

More fixational saccades were detected for Monkey E than C (see Table 2 and compare the vertical axes in Figures 3A,B). Perhaps contributing to this difference were the 46% more trials collected for Monkey E, though this cannot wholly account for the 528% more saccades. We also considered whether the proprietary heuristic filter applied online to Monkey C (compared to our own implementation applied to Monkey E; see Materials and Methods, Offline Signal Processing) contributed to the discrepancy. Analysis of additional datasets from Monkey E with the proprietary filter applied to the optical data yielded similar results with many more fixational saccades detected than in Monkey C, suggesting that the difference in fixational saccade rate may be intrinsic to the two animals. In addition, compared to our implementation, we found the proprietary filter in Monkey E reduced the proportion of saccades detected only by the optical system (from 6.8 to 3.4%) as well as those detected only by the coil (from 3.5 to 2.7%). This change suggests that the proprietary filter was more effective than our own implementation at reducing noise that may have contributed to false positive detection. (Note that we nonetheless employed our own filter implementation for all analyses since it could be applied equally to both optical and coil data, and therefore allow us to attribute differences between the systems to the techniques themselves and not subsequent signal processing—see Materials and Methods, Offline Signal Processing).

### Experiment 1, analysis 3: instructed saccades

**Figure 4:**
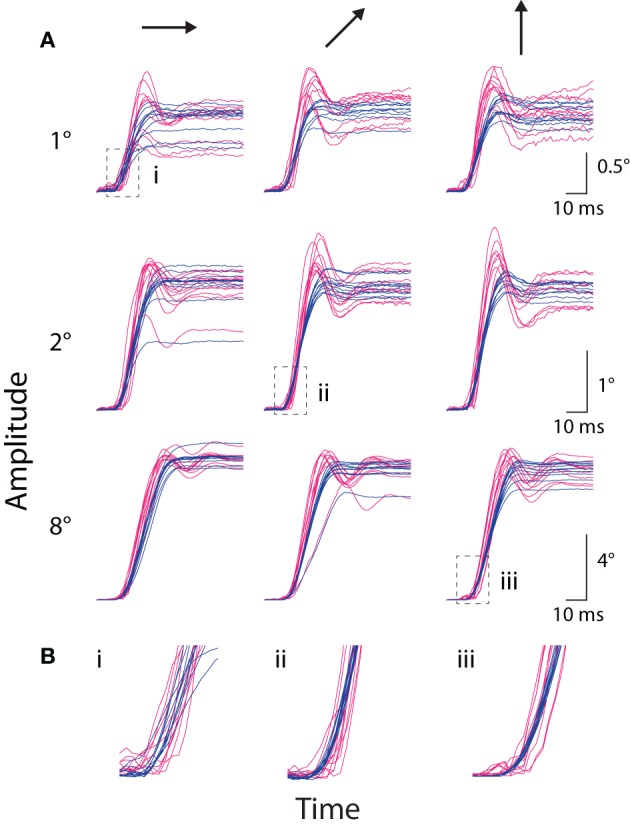
**Instructed saccade traces. (A)** The amplitude vs. time traces for all instructed saccades to each of nine representative targets from Monkey E, with panels grouped by target amplitude (*rows, top-to-bottom*: 1, 2, and 8°) and direction (*columns, left-to-right*: rightward, upward-rightward oblique (45°), and upward). Scale bars apply across each row. Traces are aligned to saccade onset as detected by the coil system. Optical traces are temporally shifted based on peak time in the cross-correlograms computed during static fixation (see text). **(B)** Enlargements of regions outlined by *boxes* in **(A)**, matched by accompanying Roman numeral. Colors as in Figure 1.

In addition to fixational saccades, we compared the systems' measurement of stimulus-driven, instructed saccades to a peripheral target (e.g., Figures 1C,D). In Figure 4, we plotted amplitude traces of all saccades to several representative targets, with traces aligned to the time the coil system detected the saccade. Examination of these individual saccade traces revealed several key qualitative features. First, the rising and high-velocity phases of traces from both systems were remarkably stereotyped at the single saccade level, suggesting a high degree of precision in saccade measurement (alignment to the coil-detected saccade time contributed to apparent temporal variability in the optical traces). Second, variation in the final eye position was evident across trials; in the more extreme cases, both systems reported similar outlier values (e.g., 2° rightward and 8° oblique), suggesting the systems accurately detected true underlying variation in saccade amplitude. Third, the initial rise in amplitude often appeared earlier in the coil than optical systems. Fourth, traces from the optical system were systematically steeper in their high-velocity phase than those from the coil system, indicating higher peak-velocities in the optical measurements. Fifth, at the conclusion of many saccades, the optical system reported an overshoot—in which eye position exceeded the final resting position—that was typically followed by additional phases of oscillation about the final eye position. This phenomenon was less common in the coil system, and when it did occur, typically consisted of a single phase. We collectively term this phenomenology *ringing*. We next explore the above and other observations quantitatively.

#### Saccade metrics

**Figure 5:**
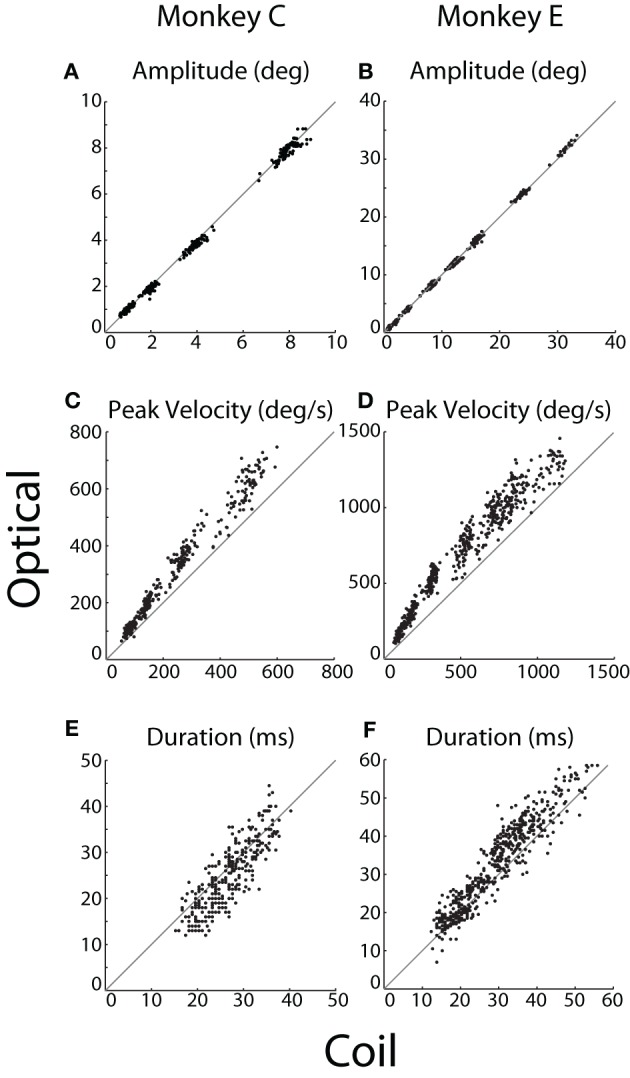
**Saccade kinematics.** For each instructed saccade (*dots*), **(A,B)** amplitude, **(C,D)** peak velocity, and **(E,F)** duration were measured by the optical system (*ordinate*) and plotted against that measured by the coil system (*abscissa*). The unity line is represented in *gray*. Data from Monkeys C and E shown in the left and right columns, respectively.

We compared instructed saccades along several standard metrics, including amplitude, peak velocity, and duration (Figure 5). These analyses included saccades made from the central FP to a peripheral target and, for Monkey E, saccades made from the top-right corner (Materials and Methods). Amplitude refers to the distance between eye position at saccade onset and offset, where offset was marked at the conclusion of any post-saccadic ringing. We measured peak velocity as the maximum speed during the primary eye movement, before any ringing occurred. Duration was defined as the difference in time between saccade offset and onset. If any ringing were detected, the time of saccade offset was taken as the middle of the ringing period (defined below).

We compared the kinematics of individual saccades, as plotted in Figure 5. Measurements of saccade amplitude were nearly identical in the two systems (Figures 5A,B), differing by at most 1%. Specifically, the slopes of the linear least-squares fit to these data were 0.99 and 1.01 (difference from unity slope: *p* = 0.014 and *p* < 10^−12^) for Monkeys C and E, respectively (fits not shown). The average pairwise difference across all saccades revealed slightly greater coil amplitudes for Monkey C (0.06°, *p* < 10^−11^), with no significant difference for Monkey E. Saccade velocities, however, differed substantially between the two systems (Figures 5C,D). Peak velocities measured by the optical system were 21 or 13% greater than their coil counterparts (difference from unity slope: *p* < 10^−52^ or *p* < 10^−31^) for Monkeys C and E, respectively, as again revealed by linear fits. Absolute pairwise differences in peak velocity are less meaningful given the range of velocities measured, but these, too, showed significantly elevated optical velocities. In terms of duration, optical durations increased more rapidly than coil durations as absolute saccade duration increased (slopes of linear fit: 1.12 and 1.11; difference from unity slope: *p* = 0.002 and *p* < 10^−10^, for Monkeys C and E, respectively). However, the relationship was complicated such that for short duration saccades, coil durations tended to be longer, while the opposite was true for longer duration saccades (Figures 5E,F). The comparison of saccade durations was confounded by post-saccade ringing—particularly prevalent in the optical system and especially for Monkey E—that obscured the true endpoint of a saccade and tended to be more prevalent for larger saccades (see Ringing). The ringing likely contributed to both the elevated optical durations for longer saccades and the more pronounced elevation in Monkey E than C.

We were interested in the sensitivity of the two systems to the fine variation in saccade metrics that occurred across repeated movements to the same target. We therefore computed the correlation between optical and coil systems at each target eccentricity separately. Across all eccentricities, we found significant (*p* < 0.001) correlations in saccade amplitude (*r* > 0.74 or 0.62) and peak velocity (*r* > 0.69 or 0.76) for Monkeys C or E, respectively, except at 32°, where peak velocity was weakly but not significantly correlated (*r* = 0.37, *p* = 0.1, Monkey E only). Correlation between the duration measurements was smaller and often non-significant in both animals, again likely due to distortion of our measurement by the post-saccadic ringing.

In addition to the metrics above, we compared the onset time of the instructed saccades. As observed qualitatively in Figures 4A and especially 4B, the initial change in amplitude in the optical traces appeared to lag that in the coil traces. (Traces were aligned to saccade onset in the coil system, contributing to the perceived increase in temporal variation in the optical traces). Indeed, saccades in the optical system were detected on average 0.6 ms later than in the coil system (*p* < 10^−17^) for Monkey E (no significant lag was observed for Monkey C). Differences in the dynamics of the positional traces likely explain the later saccade onset times, as discussed below. Delays in the synchronization pulse between the two systems do not contribute to saccade onset difference since we adjusted the relative timing of the traces for the average lag between the two systems (using the peak time in the cross-correlograms computed during static fixation—see above and Figures 2K,L).

We confirmed that instructed saccades from both systems conformed to the well-known main sequence (Bahill et al., 1975b), with established formulae (Yarbus, 1967; Becker, 1989) explaining well the relationship between amplitude and peak velocity (Monkey C: *R*^2^_coil_ = 0.55, *R*^2^_optical_ = 0.49; Monkey E: *R*^2^_coil_ = 0.91, *R*^2^_optical_ = 0.91) and between amplitude and duration (Monkey C: *R*^2^_coil_ = 0.78, *R*^2^_optical_ = 0.82; Monkey E: *R*^2^_coil_ = 0.92, *R*^2^_optical_ = 0.91). In addition, this analysis confirmed higher peak-velocities reported by the optical system for saccades of the same amplitude in both animals (as above, Figures 5C,D).

#### Ringing

**Figure 6:**
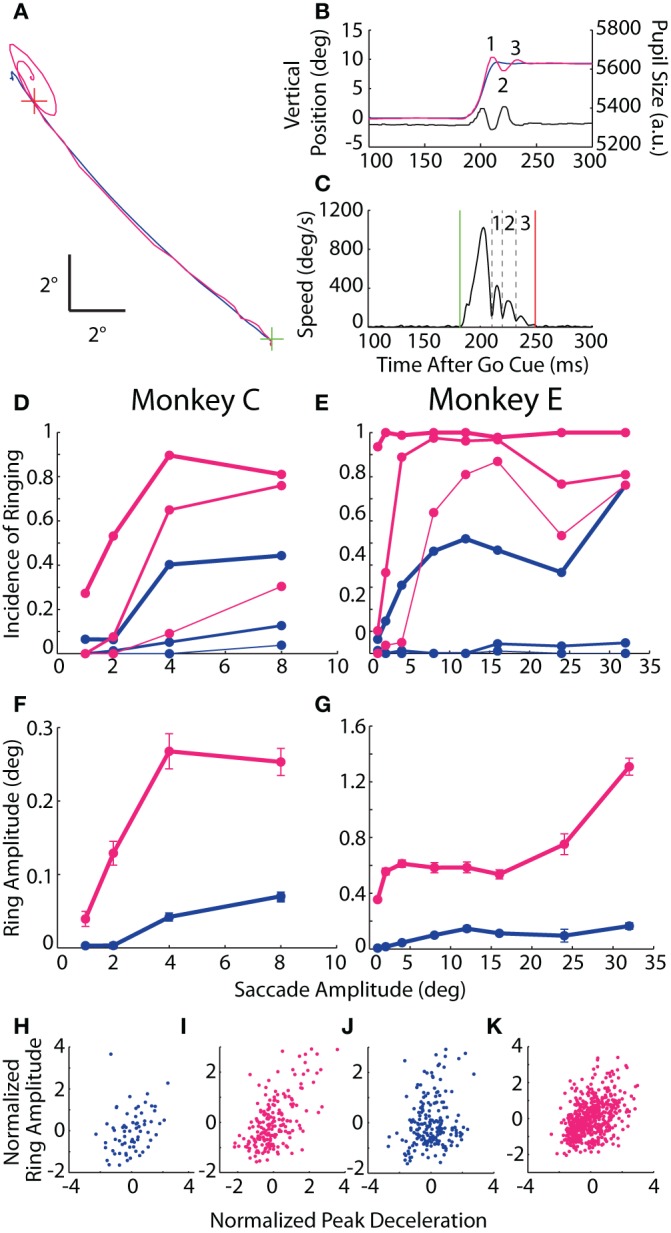
**Post-saccadic ringing. (A)** Example 12° saccade from Monkey E plotted in the horizontal-vertical plane from the central fixation point (FP; *green cross*) to target (*red cross*) demonstrating the elliptical, oscillatory trajectory at the end of the optical trace relative to the subtle overshoot in the coil trace. **(B)** Vertical position vs. time of the same saccade as in **(A)** with the individual ring phases *numbered*, and showing the coincidental oscillation in pupil size. **(C)** Speed profile from the optical measurement of the saccade in **(A,B)** demarking the saccade onset (*green line*), offset (*red line*), and intervening minima in eye speed coupled with a change in direction (*black dashed lines*) that are used to delineate the phases of ringing (*numbered* as in **B**). **(D,E)** The proportion of saccades with at least *N* phases of ringing is plotted against saccade amplitude, where *N* = 1, 2, or 3 (*thick*, *medium*, or *thin lines*). **(F,G)** For saccades with ringing, mean amplitude of the first phase is plotted against saccade amplitude. Error bars represent SEM. **(H–K)** Normalized ring amplitude is plotted against normalized peak deceleration for individual ring-containing saccades (*dots*). Both metrics are normalized within a given target eccentricity and then pooled across targets. Data from Monkeys C and E are shown in panels **(D,F,H,I)** and **(E,G,J,K)**, respectively. Colors as in Figure 1.

As observed in the example trial (Figures 1C,D at 125 ms) and in the saccade traces in Figure 4, the conclusion of many saccades was accompanied by an oscillatory process, or ringing, in which the reported position deviated about the final resting position of the eye (Figures 6A,B), often accompanied by a simultaneous oscillation in the pupillary trace. To quantify the ringing phenomenon, we delineated each eye movement trace into discrete *phases* by detecting local minima in speed that coincided with a reversal in the direction of movement (Figure 6C). (The primary eye movement, or “zeroeth” phase, was excluded from ringing analysis). The *ringing period* spanned the onset of the first phase to the offset of the final phase, or when eye velocity fell below our detection threshold. For each phase, *ring amplitude* was measured as the distance from the position at the start of the phase (i.e., trough in the speed profile) to the post-saccadic position at the end of the ringing period.

In both animals, ringing of at least one phase was observed in the vast majority of saccades recorded by the optical system (nearly 100% incidence for Monkey E), while, in the coil system, a single overshoot was detected in roughly 40% of saccades with amplitudes of at least 4° (Figures 6D,E). The incidence of additional phases of ringing also increased with saccade amplitude, and, though common in the optical system (65–90% incidence for ≥4° saccades), was rare in the coil system (<10%). In terms of ring amplitude (Figures 6F,G), we found in both systems that the amplitude of the first phase increased with the size of the instructed saccade (Monkey C: *F* = 14.0, *p* < 10^−3^; Monkey E: *F* = 43.7, *p* < 10^−10^), though the amplitude of ringing was greater in the optical system (Monkey C: *F* = 11.9, *p* < 10^−3^; Monkey E: *F* = 43.2, *p* < 10^−10^) and, in Monkey E only, increased more severely with increasing saccade amplitude than in the coil system (*F* = 17.7, *p* < 10^−4^). Importantly, we observed that pupil size oscillated in phase with the positional ringing of the optical system (e.g., Figure 6B). The dynamics of the pupil size oscillation were much faster than observed during fixation, even with rapidly changing luminance, and thus likely represented an artifactual interaction between pupil size and position that we explored below. The ringing phenomenon was not due to the Ellipse pupil fitting method (see Materials and Methods); we observed similar results when using the Centroid method with Monkey E in a separate dataset.

If the ringing were intrinsic to the eye movement (rather than a measurement artifact peculiar to either system), then we would expect ringing to occur coincidentally in both systems more than in either system alone. Indeed, in Monkey C, both systems detected ringing in 19.9% of all saccades, which was significantly more than the 15.3% one would predict if the ringing occurred independently across systems (χ^2^ = 15.1, *p* < 10^−4^). In Monkey E, ringing occurred in 99% of all optically tracked saccades, thus making a statistical comparison difficult; nonetheless, both systems detected ringing in 35.1% of all saccades, which was marginally greater than the 34.6% predicted by chance (χ^2^ = 3.8, *p* = 0.0504).

Given that the incidence and amplitude of ringing increased with saccade amplitude in both systems, we considered a physiological explanation for the phenomenon, that is, an oscillatory movement of the globe and/or internal ocular structures. We elaborate on a specific mechanism in the Discussion. In brief, we considered whether the optically tracked ringing resulted from the rapid deceleration of an elastic system (i.e., the iris suspended in the globe), much in the way an automobile might rock back and forth on its suspension after coming to an abrupt halt. Similarly, the coil-detected ringing might result from an over-rotation of the globe beyond its intended target that is subsequently corrected (Bahill et al., 1975a; Van Gisbergen et al., 1981), a phenomenon that may be more common for more rapidly decelerating movements whose control is less precise. Though different physical mechanisms may underlie the ringing in each system, we would predict that those saccades undergoing greater deceleration should result in greater amplitude of post-saccadic ringing. To test this prediction, we computed the correlation between peak deceleration and amplitude of the first phase of ringing (given that ringing was detected) separately for each system. Since peak deceleration covaries with saccade amplitude, we first normalized both measures within target eccentricity, while excluding eccentricities with fewer than 10 ring-containing trials (Figures 6H–K). For both animals and both systems, we found a significant positive correlation between saccade peak deceleration and amplitude of ringing (Monkey C *r*_coil_ = 0.31, *p* = 0.011; *r*_optical_ = 0.59, *p* < 10^−58^; Monkey E *r*_coil_ = 0.2, *p* = 0.006; *r*_optical_ = 0.41, *p* < 10^−22^).

While the above analysis controlled for a gross influence of saccade amplitude (by normalizing within target eccentricity), we went a step further to isolate the influence of saccade peak deceleration above and beyond that attributable to trial-to-trial variation in saccade amplitude and peak velocity (even within a target eccentricity). We constructed a multiple linear regression model of first-phase ring amplitude with regressors of saccade amplitude, peak velocity and peak deceleration, all normalized within target eccentricity. We found a significant, positive influence of peak deceleration on ring amplitude in both animals for the optical system and in Monkey E for the coil system (β = 0.184–0.206, *p* < 0.05). The coil data from Monkey C, which had the fewest number of saccades with ringing, showed a comparable influence of peak deceleration (β = 0.185), but was not significant.

### Experiment 1, analysis 4: trial-to-trial drift

**Figure 7:**
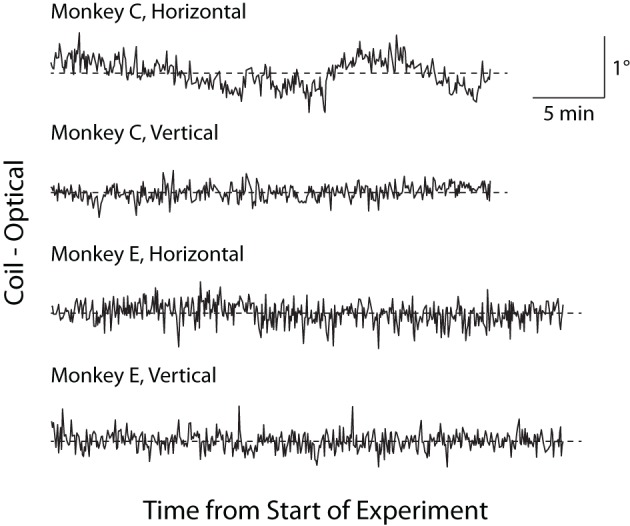
**Trial-to-trial drift over experimental session.** Difference in fix-period eye position between the two systems (coil–optical) on each trial is plotted over the course of an experiment separately for horizontal and vertical dimensions. *Dashed lines* represent zero difference.

Historically, a concern with optical tracking systems has been *trial-to-trial drift*, or an artifactual, systematic change in reported eye position unfolding over minutes to hours (van der Geest and Frens, 2002). This trial-to-trial drift is distinguished from the more rapid change in eye position that may occur within a trial (see Luminance/Position Interaction below). To test for trial-to-trial drift, we linearly regressed the average horizontal and vertical eye positions reported by the two systems in the final 300 ms of the fix period against time within an experimental session. In Monkey C, we found small but significant drift in the coil (horizontal = −0.204°/h, *p* = 0.015; vertical = 0.282°/h, *p* < 10^−6^) and optical systems (horizontal = 0.193°/h, *p* = 0.016; vertical not significant), while in Monkey E, we observed a small degree of drift only in the coil system (horizontal = −0.155°/h, *p* < 10^−9^; vertical not significant) with no significant drift in the optical system. To control for a common contributor to the drift (such as a systematic change in veridical eye position), we plotted the difference between the systems (coil–optical) vs. time in Figure 7 and applied a linear fit to this relationship. The results of this difference analysis were consistent with the previous analysis, with small but significant drift in Monkey C (horizontal = −0.397°/h, *p* < 10^−6^; vertical = 0.206°/h, *p* < 10^−6^) and Monkey E (horizontal = −0.223°/h, *p* < 10^−5^; vertical not significant). Overall, however, we note the extremely small magnitude of the drift (at most 0.3° over the course of an hour for any given system) that, if anything, appears more common in the coil than optical systems. Moreover, upon visual inspection, the scale of any systematic drift is considerably less than the gross fluctuations in position from trial to trial.

### Experiment 2, analysis 1: luminance/position interaction

**Figure 8:**
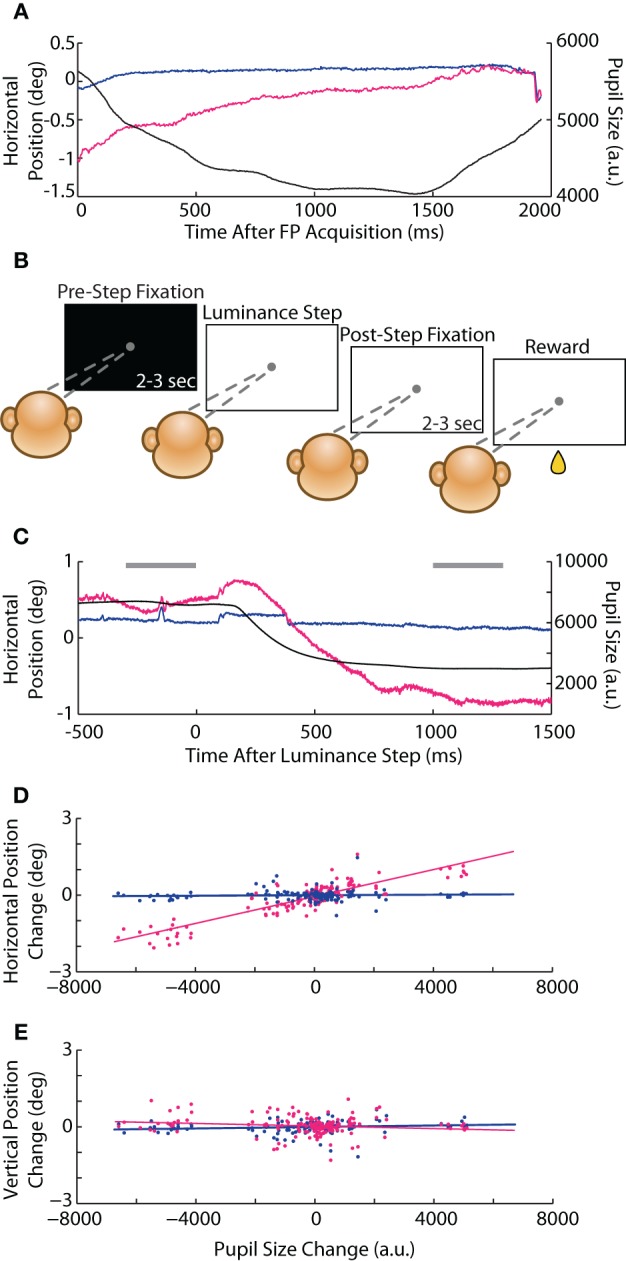
**Luminance-step task and pupil size-position crosstalk. (A)** Example horizontal position and pupil size traces from Monkey C in the instructed saccade task showing slow, coincidental changes in pupil size and optically measured eye position after foveation of the central fixation point (FP). **(B)** Schematic of the *vary-BG* version of the luminance-step task used to measure size-position crosstalk (Monkey E only). The animal fixated a gray circular FP displayed against either a white or black background. After maintaining fixation for 2–3 s (*pre-step* period), the background luminance changed to one of five shades of gray (*step*). After additional fixation of 2–3 s (*post-step*), the animal received a drop of juice (*reward*). In the *vary-FP* version (not shown), FP luminance changed at the step while the background remained constant. **(C)** Example *vary-BG* trial in which an increase in brightness at the step (*time* = 0) induced pupillary constriction and coincidental change in horizontal eye position in the optical system. *Horizontal gray bars* demark time periods used for computing pre- and post-step pupil size and position. **(D,E)** For each trial, pre- to post-step change in horizontal **(D)** and vertical **(E)** eye position are plotted against change in pupil size (*dots*) for both systems and pooled across vary-BG and vary-FP tasks, with *lines* representing least-square fits. Colors as in Figure 1.

In our initial instructed saccade experiments, we observed a possible interaction between pupil size and eye position that manifested as a rapid change in reported eye position during fixation. For instance, when the animal first foveated the FP at the start of a trial, the abrupt increase in luminance triggered a reflexive constriction of the pupil that was accompanied by a coincidental change in eye position reported only by the optical system (e.g., Figure 8A), ranging from 0.2 to 1° in amplitude and following the timecourse of the pupillary constriction (~1 s). (Positional change was less apparent upon foveation of the subsequent saccade target, which did not involve as drastic a luminance change—see Figure 1D, vertical trace at 200 ms and compare magnitude of pupillary and positional changes, as well as timescale to Figure 8A). To test this potential size-position crosstalk explicitly, we developed a luminance-step task (see Materials and Methods, Experiment 2) in which the animal (Monkey E only) foveated a static FP while we systematically varied the background (BG) or FP luminance during fixation (Figure 8B). We quantified the size-position relationship as the change in eye position (both systems) vs. change in pupil size (measured with the optical system) from the period before (*pre-step*) to after (*post-step*) the instantaneous luminance change. The average pre-step position and size were taken from the 300 ms preceding the luminance step, while the post-step measurements were taken from a 300 ms epoch starting 1 s after the step, which is when we generally observed the conclusion of any pupil size change (e.g., Figure 8C). The slope of the linear least-squares fit to the size-position relationship quantified the magnitude of crosstalk; we performed the calculation independently for horizontal (Figure 8D) and vertical (Figure 8E) eye position, and eventually pooled across the vary-BG and vary-FP trials prior to fitting, as in the figures.

In the vary-BG experiment, we introduced a step-change in the background luminance while the animal maintained fixation; in the vary-FP experiments, we instead introduced a step-change in the FP luminance, producing luminance changes perhaps more akin to those that would occur within a trial as the subject saccaded between stimuli of differing luminance. As expected for the optical system, the absolute changes in pupil size and position were greater in the vary-BG than vary-FP tasks (2° vs. 0.5° maximum change in position). However, we found no significant differences in the extent of crosstalk, or slope, between the two tasks (vary-BG vs. vary-FP: horizontal 2.64 * 10^−4^ vs. 2.48 * 10^−4^°/a.u., *p* = 0.69; vertical −2.89 * 10^−5^ vs. 5.17 * 10^−6^°/a.u., *p* = 0.50); we therefore combined data across the two tasks for subsequent analysis. In the combined dataset (Figures 8D,E), we found a significant position-pupil interaction only for the horizontal dimension in the optical system (slope = 2.63 * 10^−4^°/a.u., *p* < 10^−50^), whereas position in the vertical dimension and both dimensions in the coil system were not influenced by changes in pupil size.

We addressed two potential factors that may have influenced the size-position interaction. First, we were concerned that occlusion of the pupil may lead to a size-position interaction, which may occur when a sudden decrease in luminance maximally dilates the pupil. We reanalyzed the luminance-step data after removing trials that involved a screen luminance dimmer than used for the instructed saccade task, which was calibrated to prevent pupillary occlusion. However, even when omitting trials with maximally dilated pupil sizes, we continued to observe a significant size-position interaction for the horizontal dimension (slope = 3.05 * 10^−4^°/a.u., *p* < 10^−5^). Second, to control for the pupil fitting method, we repeated the luminance-step experiment with the alternative Centroid method. Here we found a similar degree of crosstalk in the horizontal dimension (slope = 2.77 * 10^−4^°/a.u., *p* < 10^−48^), but additionally found crosstalk in the vertical dimension, as well (slope = −1.39 * 10^−4^°/a.u., *p* < 10^−26^). The significant vertical crosstalk, present only in the Centroid data, may be attributable to partial occlusion of the dilated pupil by the eyelid, to which the Centroid method is more sensitive. We therefore reanalyzed the Centroid data, again removing trials in which the pupil was likely occluded. However, we again found a significant size-position interaction in both the horizontal (slope = 1.22 * 10^−4^°/a.u., *p* = 0.043) and vertical (slope = −1.59 * 10^−4^°/a.u., *p* = 0.010) dimensions. Therefore, whether the pupil size-position interaction involves the vertical channel depends on the pupil tracking method used and is not simply explained by pupillary occlusion.

Finally, we attempted to correct for the size-position interaction online using an undocumented parameter setting provided by the manufacturer that purportedly would back-out the spurious change in position based on the empirically measured extent of interaction. However, these efforts did not influence luminance-induced deviations in reported position. (A prior report of luminance effects on optical tracking of a prosthetic eye (Traisk et al., 2006) focused on directionless electromagnetic noise, and is thus unrelated to the directional errors in eye position linked to pupillary changes that are of concern here).

## Discussion

We have compared the suitability of the coil and optical eye tracking systems for various applications. Our results demonstrate broad agreement between the systems, and yet they do differ in several key respects. Here we summarize the similarities and differences, especially as they pertain to typical applications and, in the case of discrepancies, we consider their underlying causes.

Both systems accurately localize eye position to within 0.1° at central eye positions, or to 0.6° at very eccentric positions, in accord with the informal consensus among several laboratories that have used both systems extensively. This coarse accuracy across the oculomotor field is particularly important in free-viewing or visual search studies when knowledge of the exact stimulus being foveated is desired (e.g., Yarbus, 1967; Bichot et al., 2005), in behavioral studies when multiple response targets are arrayed, and in visual or oculomotor studies of peripheral response fields that require fixation of an eccentric FP. We note that the accuracy of the coil system declines more rapidly than the optical system as target eccentricity increases, albeit slightly (~0.2°), and particularly in the corners where saturation is greater (Figure 1B). This difference is mitigated by the biquadratic offline calibration (corner inaccuracies are marginally worse with the linear online calibration alone). In our experience, this trend is not uncommon with other coil systems—including several physically distinct systems of varying field strength in our own laboratory—and should be considered if a high degree of accuracy at very eccentric positions is required.

We distinguish coarse accuracy from finer variation in recorded eye position that likely occurs across time and between trials when fixating a static target. We assessed this fine variation by correlating position signals between the systems, reasoning that positional changes detected by both systems likely represented true variation. Here we were impressed by the degree of correlation (but see the caveat below regarding the size-position interaction in the optical system interfering with detection of slow fixational drift movements). For instance, despite trial-to-trial variation at the central FP of less than 0.2°, roughly 25% of this spread represented true variation in eye position. In a more difficult test, the sample-to-sample variation within a short epoch of fixation (and excluding known saccadic movements) was also correlated, albeit very weakly (*R*^2^ = 1.2%), despite very small variation in these periods. Though our results do not allow for a completely unambiguous measure of accuracy at these fine scales, the systems appear to be sensitive to variations in eye position of at least 0.2° (though not *all* variation on this scale is veridical).

Despite the correlation in eye position, the systems differed in the absolute magnitude of variation reported, which we take as a metric of precision. Within a brief period of fixation (300 ms), the reported eye position varied 0.04–0.1° for the optical system, while only 0.01–0.03° for the coil. At longer timescales, the greater variation in the optical system became more pronounced (saturating at 0.12–0.16° vs. 0.04° for the coil system), likely due to luminance-induced positional change (discussed below) as well as unknown mechanisms that cause a slow oscillation, or wobble, in the optical traces (Drewes et al., 2011).

Measures of sensitivity and precision are perhaps more relevant than absolute position in certain applications. For instance in neurophysiological studies of neurons with small receptive fields (e.g., 0.5° in primary visual cortex), the experimenter must determine if a difference in neural response, say across trials, is due to an intended change in the visual stimulus, or due to a small, unintended difference in eye position. Likewise, small dynamic changes in eye position can introduce neural responses in, say, motion-sensitive neurons (e.g., Bair and O'keefe, 1998; Hohl and Lisberger, 2011), thus requiring sensitivity of the tracker to fine, rapid eye movements. In selecting an eye tracking system, one should consider the sensitivity of the experimental system under study to fine changes in eye position (e.g., response field size) and the duration for which one requires precise positioning.

When detecting fixational saccades, the burden of precision is compounded by a need for adequate temporal resolution to capture small and rapid changes in eye position. Remarkably, at least 90% of saccades detected by one system were also detected by the other system, and the missed saccades were of very low amplitude (median 0.15–0.17°). The range over which both systems detected fixational saccades (>0.1°) overlaps with that of so-called microsaccades (Cornsweet, 1956; Zuber et al., 1965; Steinman et al., 1973; for review, Martinez-Conde et al., 2004), thus allowing for study of this special class. In practice, we recommend use of a heuristic filter for noise-suppression of the optical data as described above; in particular, the proprietary filter supplied with the present optical tracker is likely superior to the published rendition we implemented (Materials and Methods) and is capable of filtering online.

Finally, we consider the measurement of larger, instructed saccades. Both systems reported nearly identical amplitudes across a range of 1–32° saccades. Moreover, the variation we observed in amplitude and peak velocity for saccades to the same target was highly correlated between the systems, suggesting both systems are sensitive to this finer variation from movement to movement.

In one marked discrepancy, the optical system measured higher peak velocities for saccades of the same amplitude, consistent with previous studies comparing optical (including dual-purkinje image trackers) and contact lens-based coil systems (Deubel and Bridgeman, 1995a; Discenna et al., 1995; van der Geest and Frens, 2002; Traisk et al., 2005; Drewes et al., 2011). Studies using the two tracker technologies serially have suggested that the scleral coil may apply an artificial load that slows the eye movement, or that slippage of a contact lens-based coil introduces a low-pass filter on eye velocity (Traisk et al., 2005). However, our study does not support these theories since our simultaneous measurements demonstrated elevated optical peak velocities in a coil-implanted eye, and the coil was firmly embedded in the sclera. Also, previous concerns about low optical sampling rates (van der Geest and Frens, 2002) do not explain the discrepancy as eye position data from the optical system was sampled at 1 kHz, providing temporal resolution comparable to the coil system.

An alternative explanation for the elevated peak velocities hinges on the exact structures being tracked by the two systems. The coil is embedded in the sclera and thus sensitive to the position of the globe, while the optical system tracks the position of the pupil—a space defined by the iris, which itself is non-rigid. Previous studies that have optically tracked the lens (via its Purkinje image), have proposed that the elastic nature of the lens' attachment allows it to remain still as the globe begins to rotate; then, at some delay, the elastic zonular fibers attached to the lens recoil, tugging the lens into flight at a higher velocity than the globe itself. Inhoff and Radach (1998) have suggested that a similar spring-like mechanism may apply to the pupil, allowing it to move independently of the globe. Consistent with this theory, we observed a relative lag in the initial movement in the optical traces that was then followed by a higher peak velocity, such that the optical trace ultimately rejoined and typically passed that of the coil (Figure 4).

A similar mechanism may operate at the end of saccades, thus explaining the ringing phenomenon so prevalent in the optical traces. Specifically, the globe may stop rotating abruptly, while the pupil decelerates more gradually, overshooting and oscillating about its final position before coming to rest—a mechanism first proposed to explain ringing of the lens (Deubel and Bridgeman, 1995a). Consistent with such a mechanism, the degree of deceleration we observed at the single-saccade level predicted the amplitude of post-saccadic oscillation beyond that predicted by saccade amplitude or peak velocity. Notably, optically tracked saccades in Monkey C did not show a significant lag in onset and coincidentally did not exhibit as great an incidence or amplitude of post-saccadic ringing as in Monkey E, perhaps due to a less elastic pupil. In summary, a physiological basis—movement of internal ocular structures independently of the globe—may underlie the post-saccadic ringing. Interestingly, these post-saccadic oscillations have been associated with perceptual consequences, further suggesting their veridicality (Deubel and Bridgeman, 1995b).

Considerably less ringing was observed in the coil traces, and virtually always was limited to a single phase. Given the discrepancy between systems, one is tempted to ask which system more accurately represents post-saccadic eye position. Prior studies report occasional multiphasic oscillations measured by the coil system, even for microsaccadic movements, that likely represent a brainstem-mediated dynamic correction process (Van Gisbergen et al., 1981). Therefore, the present discrepancy between systems may be due to reduced sensitivity of the coil system in our hands to fine oscillations of the globe. However, this seems unlikely given that the coil system was capable of detecting smaller fixational saccades, measured smaller oscillations when post-saccadic ringing was detected, and reported less noise during static fixation. Alternatively, as discussed above, it is possible that both techniques are accurate, only they each represent the position of different ocular structures (globe vs. iris). Perhaps the more practical question then would be which system, and hence which ocular structure, more closely predicts a subject's percept, a topic deserving of further study.

In a separate luminance-step task, we directly tested the tendency of the optical system to mistake changes in pupil size for changes in position. While this confound could be large for extreme changes in luminance, it was generally modest for the luminance range of a typical task. In practice, we found the extent of size-position crosstalk was further mitigated by increasing background luminance (thus reducing the percent luminance change across different fixation and saccade targets), equating luminance of the targets themselves, and allowing adequate time for the pupil to adapt to the initial luminance change at the beginning of a trial (crosstalk was more pronounced upon acquiring the FP than subsequent targets). Curiously, the extent of luminance-induced crosstalk during fixation was more pronounced in Monkey E (qualitatively from reviewing fixation traces, since Monkey C did not perform the luminance-step task), for whom the overall sample-to-sample variation was also greater (as expected with increased crosstalk), thus suggesting a possible subject-dependence of the luminance confound. We do not believe the size-position crosstalk should be a limiting factor except in those task designs that require large changes in luminance coupled with relatively fine (<1°) estimates of position on the timescale of pupillary changes. In particular, study of fixational drift movements that unfold over ~0.5 s and span ~0.1° (for review, Martinez-Conde et al., 2004; Poletti et al., 2010) may preclude use of optical tracking for these reasons and would benefit from techniques that are insensitive to pupillary changes, such as coil- or Purkinje image-based systems.

Previous reports have voiced concern regarding the stability of optical signals over the course of an experiment (van der Geest and Frens, 2002). Our present data suggest this trial-to-trial drift should no longer be a concern, with changes of at most 0.2°/h, comparable to or less than that observed with the coil system.

While our comparison focused on fixation of and saccades to static targets by tracking a single eye, several important classes of eye movements were not studied. In particular, we did not examine smooth pursuit eye movements, for which a direct comparison is certainly warranted. However, we note that our study included eye velocities of less than (fixation) and greater than (saccades) those found in smooth pursuit, and did so on very fine spatial scales (~0.1°); therefore, we would predict comparable performance on smooth pursuit tracking as reported here for fixation and saccade tracking. Also absent from our study was a binocular comparison of conjugate and vergence movements.

Our results provide the first comparison of the sclera-embedded search coil and infrared optical tracking systems recorded simultaneously in the same primates. The optical technique has improved considerably within the past decade, and our study shows that optical systems can now rival the search coil for all but the most exacting applications. With the data provided, we hope laboratories around the world will be able to judge exactly which technique is best for their applications, human and non-human studies alike. This decision is of substantial consequence, since widespread adoption of the optical systems could reduce the number of invasive surgeries required for rigorous study of eye position and movement.

### Conflict of interest statement

The authors declare that the research was conducted in the absence of any commercial or financial relationships that could be construed as a potential conflict of interest.
